# Dynamic Temporal Denoise Neural Network with Multi-Head Attention for Fault Diagnosis Under Noise Background

**DOI:** 10.3390/s24216813

**Published:** 2024-10-23

**Authors:** Zhongzhi Li, Rong Fan, Jinyi Ma, Jianliang Ai, Yiqun Dong

**Affiliations:** Department of Aeronautics and Astronautics, Fudan University, Shanghai 056004, China; zzli22@m.fudan.edu.cn (Z.L.); rfan24@m.fudan.edu.cn (R.F.); jyma21@m.fudan.edu.cn (J.M.); aijl@fudan.edu.cn (J.A.)

**Keywords:** nonlinear denoising, multi-time resolution decomposition, short-time fourier transform, mixed precision calculation, fault diagnosis

## Abstract

Fault diagnosis plays a crucial role in maintaining the operational safety of mechanical systems. As intelligent data-driven approaches evolve, deep learning (DL) has emerged as a pivotal technique in fault diagnosis research. However, the collected vibrational signals from mechanical systems are usually corrupted by unrelated noises due to complicated transfer path modulations and component coupling. To solve the above problems, this paper proposed the dynamic temporal denoise neural network with multi-head attention (DTDNet). Firstly, this model transforms one-dimensional signals into two-dimensional tensors based on the periodic self-similarity of signals, employing multi-scale two-dimensional convolution kernels to extract signal features both within and across periods. Secondly, for the problem of lacking denoising structure in traditional convolutional neural networks, a temporal variable denoise (TVD) module with dynamic nonlinear processing is proposed to filter the noises. Lastly, a multi-head attention fusion (MAF) module is used to weight the denoted features of signals with different periods. Evaluation on two datasets, Case Western Reserve University bearing dataset (single sensor) and Real aircraft sensor dataset (multiple sensors), demonstrates that the DTDNet can reduce the useless noises in signals and achieve a remarkable improvement in classification performance compared with the state-of-the-art method. DTDNet provides a high-performance solution for potential noise that may occur in actual fault diagnosis tasks, which has important application value.

## 1. Introduction

Fault diagnosis of mechanical systems (typically such as rolling bearings, aircraft sensors, etc.) has become an increasingly critical part of engineering [1,2]. Since mechanical systems often work in harsh conditions, including high temperature, high humidity, and overload, which are prone to failure [3]. These failures can cause economic losses and safety risks and even lead to casualties [4]. Therefore, accurate monitoring and diagnosis of mechanical system faults are crucial for maintaining production safety and preventing catastrophic incidents [5].

Traditional fault diagnosis is usually based on system models or signal processing models [6,7]. Jalan et al. proposed a model-based fault diagnosis method for rolling bearing systems [8]. Dybała et al. proposed a signal processing-based diagnosis method. The pure noise part is extracted from the original vibration signal through empirical mode decomposition (EMD), and the spectral analysis method of the empirically determined local amplitude is used to further extract fault-relevant features from the pure noise signal [9]. These methods largely depend on manually identifying intricate features and heavily rely on empirical knowledge, which leads to reduced efficiency and narrow applicability of the algorithms.

Machine learning methods can automatically extract data features and have been widely applied in fault diagnosis tasks [10,11]. Typical methods include artificial neural networks (ANN), principal component analysis (PCA), K-nearest neighbors (KNN), and support vector machines (SVM), etc. Zarei et al. proposed an ANN and proximal SVM method for processing the time domain characteristics of fault signals [12]. Qian et al. used a differential evolution algorithm to preprocess time domain signals and improved the feature extraction method [13]. Islam et al. proposed a reliable multiple combined fault diagnosis method for bearings using heterogeneous feature models and an improved one-against-all multiclass SVM classifier [14]. Xue et al. proposed an improved local and global principle component analysis (LGPCA) method and used the fast ensemble empirical mode decomposition (EEMD) for multi-scale feature extraction in fault diagnosis [15]. The fault diagnosis methods based on machine learning avoid the limitations of manually identifying intricate features. However, the methods based on machine learning are often difficult to extract high-dimensional features from data due to their simple structure (fewer network layers, etc.), which limits the diagnostic performance of the algorithm.

Deep learning algorithms have more complex network structures and can learn richer feature representations from data, which have been widely used in fault diagnosis tasks [16,17,18]. Janssens et al. proposed an equipment condition monitoring model based on convolutional neural network (CNN), which automatically extracts data features [19]. Xu et al. proposed a hybrid deep learning model based on CNN and deep forest that processed signals through continuous wavelet transformation (CWT) [20]. Mao et al. proposed a new type of deep autoEncoder model (DAE) that combines various types of information, has effective land acquisition capabilities, and has simplified the DAE network structure [21]. Saucedo et al. proposed a novel data-driven diagnostic method based on stacked autoencoders for diagnosing and identifying bearing faults of different bearing technologies (such as metallic, hybrid, and ceramic bearings) in electromechanical systems [22]. Miao et al. proposed a deep feature interactive network that uses multi-source heterogeneous data (i.e., infrared thermal images and vibration signals) for fault diagnosis [23]. However, the above studies mainly focus on the design and optimization of diagnostic algorithms, overlooking the significant noise included in the collected data in practical scenarios, which has a serious impact on the performance of diagnostic algorithms.

In recent years, various methods for solving fault diagnosis in noisy environments have been widely studied. Liao et al. proposed a signal filtering and fault feature enhancement method based on reconstruction adaptive deterministic stationary subspace filtering (Rad-SSF) and enhanced third-order spectrum, and its feasibility and effectiveness were verified under different conditions [24]. Chen et al. used 2 CNNs with different kernel sizes to automatically extract signal features of different frequencies from the original data and then used long short-term memory (LSTM) to identify the fault type [25]. Compared with other intelligent algorithms, it has better diagnosis performance between the signal-to-noise ratio (SNR) of −2 dB and 10 dB. Chen et al. proposed a stacked denoising autoencoder (SDAE) based on structure adaptive adjustment for bearing fault diagnosis in noisy environments, whose performance has been verified in experiments [26]. Zhang et al. used residual learning to improve network training and conducted experiments with SNR between 0 dB and 8 dB [27]. Huang et al. proposed a bearing fault diagnosis method based on Savitzky-Golay gramian angle field (GAF) [28]. After segmenting the collected signal, the high-frequency components containing fault information are obtained through Butterworth high pass filtering, and the GAF image is obtained using an enhancement algorithm as input for the ResNet18 model for bearing fault diagnosis. Liang et al. combined wavelet transform with improved Resnet and proposed a new rolling bearing fault diagnosis method that is robust to noise [29]. Guo et al. proposed a method based on attention CNN and BiLSTM (ACNN-BiLSTM) [30]. Short-term spatial features are extracted through attention CNN, and BiLSTM is used to extract long-distance dependence information of signals. The setting range of the SNR in the experiment is 10 dB to 20 dB. Wang et al. proposes a novel attention-guided joint learning convolutional neural network (JL-CNN), which mainly includes a joint feature encoding network and two attention-based encoder networks [31]. The JL-CNN is evaluated on the wheelset bearing dataset and motor bearing dataset. However, the diagnostic performance of this method in strong noise environments is still unsatisfactory.

To further improve the diagnostic performance of deep learning models in noise environments (especially when SNR < 0), this paper proposes a dynamic temporal denoise neural network with multi-head attention (DTDNet) method. Traditional denoising methods filter the signal before using neural networks to perform diagnostic tasks, which poses a risk of information loss and the introduction of pseudofeatures. The denoise method used in this paper is automatic noise filtering, and its working principle can be understood as using TVD blocks to separate the useful and noisy parts of the original signal (binary classification). A problem waiting to be solved is that the original signal containing noise is difficult to separate in low dimensional space (such as two-dimensional space). Similar to support vector machines (SVMs), which can more easily solve classification problems in high-dimensional space, this paper maps the raw data to high-dimensional space and uses adaptive filters to perform denoising, which achieves the highest diagnostic accuracy in noisy environments. The main highlights of this paper are as follows:

(1) Efficient multi-time-resolution signal feature extraction: The short-time Fourier transform (STFT) is used to decompose the signal to obtain different frequencies and amplitudes, which is suitable for non-stationary signal processing in complex scenes. Different frequencies of signal sorted by amplitude values are stacked into 2D images, respectively, and multi-scale 2D convolution is used to extract features on the 2D image (including intra-period and inter-period features). This method is different from the single-time-resolution mapping method, which lacks in considering the changing characteristics of different signals and signals at different times.

(2) Nonlinear noise filtering: The threshold unit in the temporal variable denoise (TVD) module can dynamically and flexibly apply nonlinear processing to the signal features. This dynamic adjustment ensures optimal noise reduction across a broad spectrum of signal types and noise levels, without the need for manual intervention or preset thresholds that may not be suitable for all conditions.

(3) Multi-head attention fusion weighting: The multi-head attention mechanism is used to dynamically weight the components of the signal at different frequencies. Different from using amplitude values at different frequencies as weighting weights, each head of the multi-head attention mechanism focuses on different features of the signal, which can learn rich and diverse feature representations.

The rest part of this paper is organized as follows. Section 2 details the principles of the proposed DTDNet and the sub-modules. Section 3 conducts experimental verification. Finally, conclusions are drawn in Section 4.

## 2. Methodology and Proposed Framework

**Figure 1:**
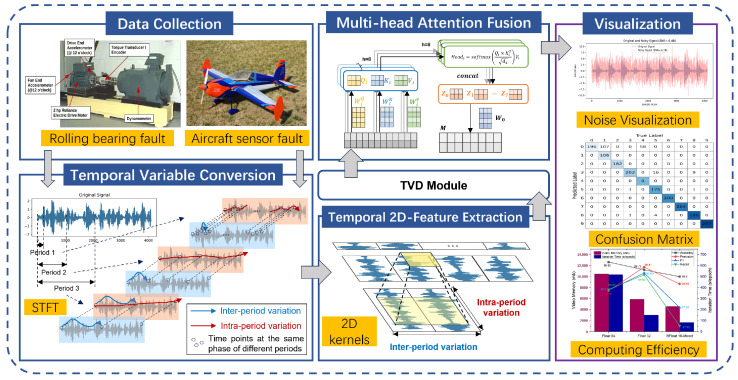
Framework of the proposed DTDNet model.

The DTDNet framework proposed in this paper is shown in Figure 1. Its core modules include temporal variable conversion (TVC), temporal 2D-feature extraction (TFE), temporal variable denoise (TVD), and multi-head attention fusion (MAF), aiming to reduce the noise in the signal and improve the classification performance of the faults. The detailed information of the data used will be introduced separately in the Results section. The individual modules of the framework will be discussed in detail in Section 2.1, Section 2.2, Section 2.3 and Section 2.4.

### 2.1. Temporal Variable Conversion and 2D-Feature Extraction Modules

**Figure 2:**
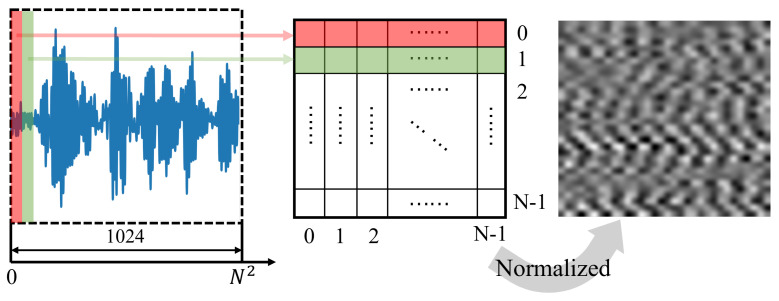
The traditional signal stacking method.

It is difficult to extract features directly from one-dimensional signals with noise [32]. A common method is to stack one-dimensional signals into two-dimensional images, which is beneficial for algorithms to extract coupling information from signals. The traditional signal stacking method is shown in Figure 2. Assuming the number of sampling points of the one-dimensional signal is $N^{2}$, the principle involves decomposing the signal into *N* segments, each with *N* sampling points, and then stacking these *N* segments into an N×N two-dimensional tensor. This stacking method can be called single-time-resolution stacking, where the value of *N* is fixed and does not consider distinctions between different signals.

**Figure 3:**
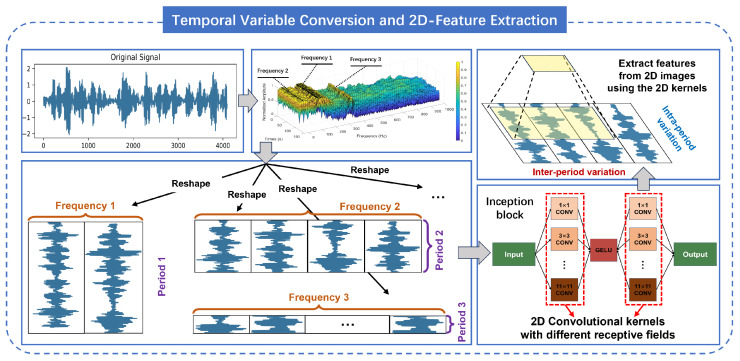
Signal stacking with multiple temporal resolutions and multi-scale feature extraction.

In order to improve the signal stacking method, this paper proposed an multi-time-resolution signal stacking method, which uses STFT to decompose the one-dimensional signal into multiple periods, and stacks the signals of different periods respectively, as shown in Figure 3.

STFT is a time-frequency domain analysis method for time-varying signals [33]. Compared with fast Fourier transform (FFT), STFT is better suited for handling non-stationary signals. Essentially, a time-limited window function, denoted as h(t), is introduced to the signal before undergoing Fourier transformation in STFT. For a signal with length *T* and sampling points *C*, its original sequence is represented as $X_{1D}\in R^{T\times C}$. In this paper, the original one-dimensional signal $X_{1D}$ is assumed to be constant within a certain short period of time, and the window function h(t) moves on the signal to convert it by segment. The calculation formula of STFT is given by Equation (1).

(1) $$STFT\left(f(t)\right)=\int_{-\infty }^{+\infty }f\left(t\right)h\left(t-\tau \right)e^{-j\omega t}dt$$

where STFT(∗) represents the calculation of STFT; f(t) is the time domain signal before transformation, $h\left(t-\tau \right)$ is the window function, and τ is the center of the window function. The calculation equations for using STFT to analyze time series in the frequency domain is shown in Equations (2)–(4).

(2) $$\begin{array}{cc} A & =Avg\left(Amp\left(STFT\left(X_{1D}\right)\right)\right) \end{array}$$

(3) $$\begin{array}{c} \;\left\{f_{1},\ldots ,f_{k}\right\}=argTopk_{f_{*}\in \left\{1,\ldots ,\left\lfloor \frac{T}{2}\right\rfloor \right\}}\left(A\right) \end{array}$$

(4) $$\begin{array}{c} p_{i}=\left\lfloor \frac{T}{f_{i}}\right\rfloor ,\;i\in \left\{1,\ldots ,k\right\} \end{array}$$

where Amp(∗) represents the calculation of amplitude value. Avg(∗) indicates that the calculated amplitude of a frequency is averaged over *C* dimensions. Due to the conjugation of the frequency domain, this paper only considers frequencies within the range $\left\{1,\;\ldots ,\;\left[\frac{T}{2}\right]\right\}$. To avoid introducing noise from high-frequency signals, this paper only selects the top *k* highest amplitude values and obtains the most significant *k* frequencies through non-normalized amplitudes $\;\left\{A_{f1},\;\ldots ,\;A_{fk}\right\}$ (where $A_{f_{n}}$ represents the intensity of the periodic basis function at frequency $f_{n}$), along with their corresponding *k* periods, where *k* is a hyperparameter. The value of hyperparameter *k* corresponds to the number of selected frequencies, and comparative experiments are conducted in the experimental section to determine its optimal value. Therefore, Equations (2)–(4) can be reformulated as

(5) $$Period\left(X_{1D}\right)=\;A,\;\left\{f_{1},\;\ldots ,\;f_{k}\right\},\;\left\{p_{1},\;\ldots ,\;p_{k}\right\}$$

Based on the selected frequencies $\left\{f_{1},\;\ldots ,\;f_{k}\right\}$ and periods $\left\{p_{1},\;\ldots ,\;p_{k}\right\}$, this paper utilizes the Equation (6) to reshape the one-dimensional time series $X_{1D}$ into two-dimensional tensors.

(6) $$X_{2D}^{i}={Reshape}_{p_{i},\;f_{i}}\left(sequence\left(X_{1D}\right)\right)$$

where sequence(∗) is to unfold the time series to adaptively fill the Reshape; $p_{i}$ and $f_{i}$ respectively represent the number of rows and columns of the two-dimensional tensor; $X_{2D}^{i}\in R^{p_{i}\times f_{i}\times C}$ denotes the two-dimensional tensor of the frequency $f_{i}$ corresponding to the one-dimensional sequence, recorded as the *i*-th tensor. With multiple different $p_{i}$ and $f_{i}$, a sequence of two-dimensional tensors $\left\{X_{2D}^{1},\;\ldots ,\;X_{2D}^{k}\right\}$ is obtained. As shown in Figure 3, the columns and rows indicate the intraperiod-variation and interperiod-variation of the signal at the corresponding frequencies.

After obtaining the sequence of two-dimensional tensors, this paper performs feature extraction by constructing an inception block containing multi-scale 2D convolutional kernels. The calculation formula is shown in Equation (7).

(7) $${\hat{X}}_{2D}^{l,\;i}=Inception\left(X_{2D}^{i}\right)$$

### 2.2. Temporal Variable Denoise Module

**Figure 4:**
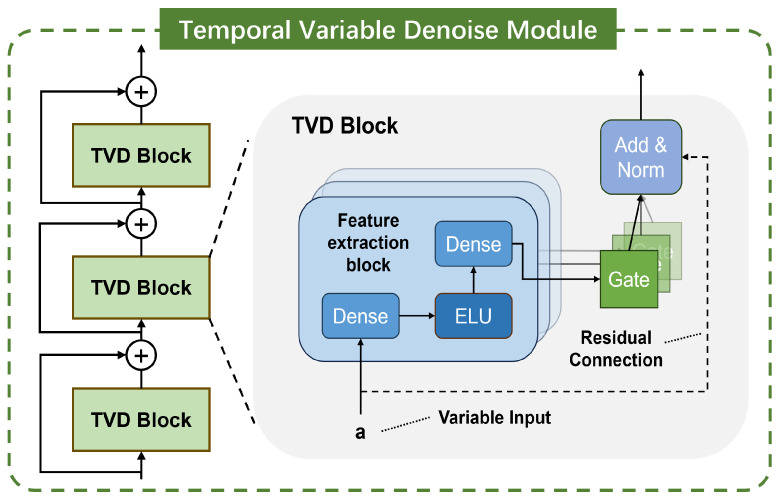
TVD module that can perform non-linear signal filtering.

In order to dynamically and flexibly reduce the noise in the input signal, the TVD module is designed in this paper, as shown in Figure 4. For the *l*-th TVD block, the computation equation is given by

(8) $${TVD}_{\omega }\left({\hat{X}}_{2D}^{l,\;i}\right)=LayerNorm\left({\hat{X}}_{2D}^{l,\;i}+{GLU}_{\omega }\left(\eta_{1}\right)\right)$$

(9) $$\eta_{1}=W_{1,\;\omega }\eta_{2}+b_{1,\;\omega }$$

(10) $$\eta_{2}=ELU\left(W_{2,\;\omega }{\hat{X}}_{2D}^{l,\;i}+b_{2,\;\omega }\right)$$

where $\eta_{1}\in R^{d_{model}}$ and $\eta_{2}\in R^{d_{model}}$ are hidden layers, and $d_{model}$ is the dimension of the hidden layers. $W_{*}\in R^{d_{model}\times d_{model}}$ is the weights, $b_{*}\in R^{d_{model}}$ is the bias, and ω represents the shared parameters of the weights. LayerNorm(∗) is standard layer normalization. ELU(∗) is the exponential linear unit activation function. When $W_{2,\;\omega }{\hat{X}}_{2D}^{l,\;i}+b_{2,\;\omega }\gg 0$, the ELU activation function will act as an identity function; when $W_{2,\;\omega }{\hat{X}}_{2D}^{l,\;i}+b_{2,\;\omega }\ll 0$, the ELU activation function will produce a constant output. ${GLU}_{\omega }\left(*\right)$ is a gating layer based on gated linear units, which functions to suppress the unnecessary parts (noise in this paper). When $\beta \in R^{d_{model}}$ is taken as input, the expression of GLU is

(11) $${GLU}_{\omega }\left(\beta \right)=\sigma \left(W_{3,\;\omega }\beta +b_{3,\;\omega }\right)⊙\left(W_{4,\;\omega }\beta +b_{4,\;\omega }\right)$$

where σ(∗) is the sigmoid activation function, and ⊙ is the Hadamard product of elements. GLU can be used to control the passage of data information flow in TVD blocks, thereby achieving the purpose of nonlinear filtering of signal. In this paper, the number of TVD blocks is set to 1.

### 2.3. Multi-Head Attention Fusion Module

${\hat{X}}_{1D}^{L,\;k}=\left\{{\hat{X}}_{1D}^{l,\;1},\;\ldots ,\;{\hat{X}}_{1D}^{l,\;k}\right\}$ is the output vector after the denoising of the TVD module (*L* is the total number of TVD blocks). This paper adopts the MAF module to dynamically weight the components of the signal at different frequencies. Different from using the amplitudes in the frequency domain as the weight, the proposed weighting method can effectively capture the critical information.

$Q_{i}$ (Query), $K_{i}$ (Key), and $V_{i}$ (Value) matrices are used to calculate the attention weights, respectively; $Q_{i}={\hat{X}}_{1D}^{L,\;k}W_{i}^{Q}$, $K_{i}={\hat{X}}_{1D}^{L,\;k}W_{i}^{K}$, and $V_{i}={\hat{X}}_{1D}^{L,\;k}W_{i}^{V}$ (*i* represent the *i*-th head). The calculation equation of the attention weights of the *i*-th head is as follows

(12) $${Attention}_{i}\left(Q_{i},\;K_{i},\;V_{i}\right)=softmax\left(\frac{Q_{i}K_{i}^{T}}{d_{attn}}\right)V_{i}$$

where $d_{attn}$ is used to scale the inner product to avoid the input of the softmax(∗) function being too large or too small. Concatenate the output vectors of multiple heads to obtain the matrix *Z*.

(13) $$Z=concat\left({Attention}_{1},\;\ldots ,\;{Attention}_{i}\right)$$

A linear transformation is applied to the matrix *Z* to obtain the weighted feature information output $h_{attn}$.

(14) $$h_{attn}=Z\times W$$

where *W* is the weight matrix that can be trained.

### 2.4. Training Strategy of the Proposed Model

**Figure 5:**
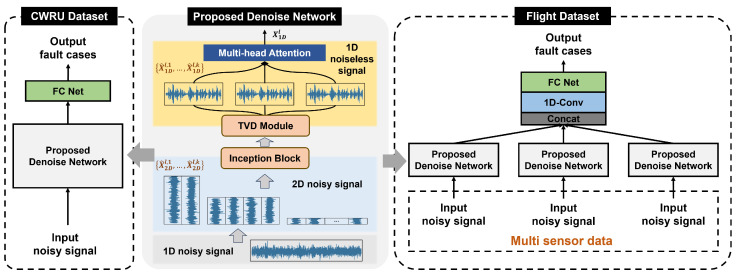
The forward propagation process of DTDNet.

The forward propagation process of the proposed DTDNet for both single-sensor and multi-sensor data during training is illustrated in Figure 5. For the rolling bearing data collected by a single sensor, the 2D images of the signal at different time resolutions are obtained after STFT decomposition. Then the inception block with the multi-scale 2D convolution kernel can be used to captured the feature information. The TVD module provides a channel for denoising the feature information. The features are weighted by the MAF module to obtain a one-dimensional tensor, which is then processed through the linear layer of the neural network to obtain the category of the fault. For real aircraft attitude measurement data with multiple sensors, the DTDNet is utilized to perform denoising on different sensors. Finally, the multiple denoised one-dimensional tensors are spliced as the input of the neural network classification layer, yielding the category of the fault.

**Figure 6:**
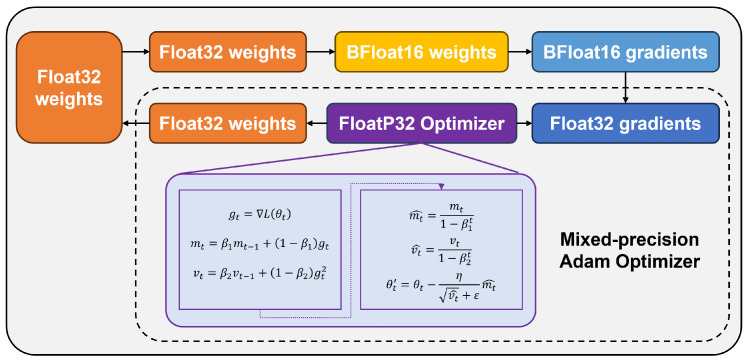
Mixed precision calculation method used by DTDNet.

To reduce computational costs, increase training speed, and save computing resource space while maintaining model performance, this paper chooses the optimization strategy of mixed precision calculation in the DTDNet model training, as depicted in Figure 6. During the forward propagation of the model training, BFloat16 precision parameters are utilized. During the backward propagation of gradients, to avoid numerical underflow caused by gradient scaling, this paper employs Adam optimizer with Float32 precision.

## 3. Results

In this section, the performance of the proposed model is verified on the dataset of Case Western Reserve University bearing data [34] and the real aircraft sensor fault [35]. The code is written on the platform of Data Science Workshop with Python 3.8. The training of the model is completed on an integrated development platform, which has one Nvidia-A100 GPU with 80 GB video memory.

### 3.1. Evaluation Metrics

Following common settings, four evaluation metrics (Accuracy, Precision, F1, and Recall) are used to evaluate the performance of the model [36]. Table 1 depicts the correspondence between the predictions of DTDNet and the true labels.

Expanding to the multi-classification tasks (taking *n* classes as an example), the values of $\vec{TP}$ (True Positive), $\vec{FP}$ (False Positive), $\vec{FN}$ (False Negative), and $\vec{TN}$ (True Negative) are *n*-dimensional vectors, where *n* represents the number of classes in the dataset (in this study, *n* = 10 and 6, respectively). In Equation (15), each dimension of the vector represents a specific value for a particular class.

(15) $$\vec{TP}=\left|\begin{array}{c} TP_{0}\\ TP_{1}\\ \cdots \\ TP_{n-1} \end{array}\right|$$

Assuming there are *M* samples, for a specific sample *S*, the true label for the *k*th ($k\in \left[0,n-1\right]$) specific class is denoted as $L_{k}$, and the predicted class is denoted as $P_{k}$.

(16) $$\begin{array}{c} L_{k}=1,P_{k}=1,\;\mathrm{then}\;{TP}_{k}=1\\ L_{k}=0,P_{k}=1,\;\mathrm{then}\;{FP}_{k}=1\\ L_{k}=0,P_{k}=0,\;\mathrm{then}\;{TN}_{k}=1\\ L_{k}=1,P_{k}=0,\;\mathrm{then}\;{FN}_{k}=1 \end{array}$$

The ${\vec{TP}}_{S}$ for sample *S* is formed by combining the aforementioned results into a vector.

(17) $${\vec{TP}}_{S}=\left|\begin{array}{c} TP_{0}\\ TP_{1}\\ \cdots \\ TP_{n-1} \end{array}\right|$$

The final result of $\vec{TP}$ is a vector obtained by summing the results for *M* samples.

(18) $$\vec{TP}={\vec{TP}}_{0}+{\vec{TP}}_{1}+\cdots +{\vec{TP}}_{M-1}$$

Using the same calculation steps and methods as the above, $\vec{FP}$, $\vec{FN}$, and $\vec{TN}$ can be obtained, respectively. The calculation formulas for evaluation metrics are as follows:

(19) $$Accuracy=\left|\frac{\vec{TP}+\vec{TN}}{\vec{TP}+\vec{TN}+\vec{FN}+\vec{FP}}\right|$$

(20) $$Precision=\left|\frac{\vec{TP}}{\vec{TP}+\vec{FP}}\right|$$

(21) $$F1=\left(2\times \frac{prec\times rec}{prec+rec}\right)$$

(22) $$Recall=\left|\frac{\vec{TP}}{\vec{TP}+\vec{FN}}\right|$$

### 3.2. Case Western Reserve University Bearing Dataset

**Figure 7:**
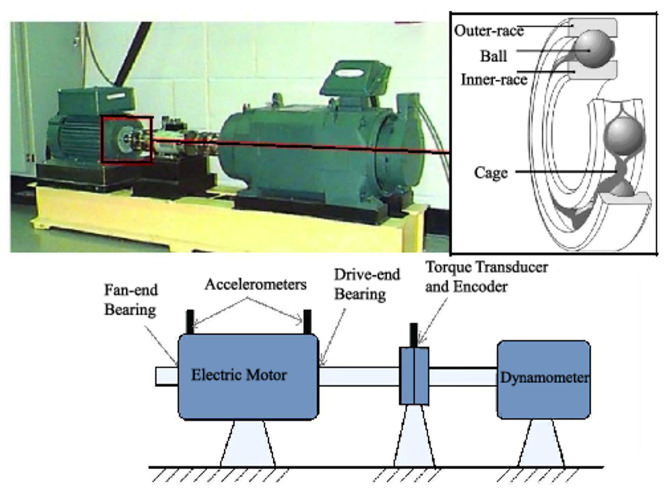
CWRU rolling bearing test platform [34].

**Figure 8:**
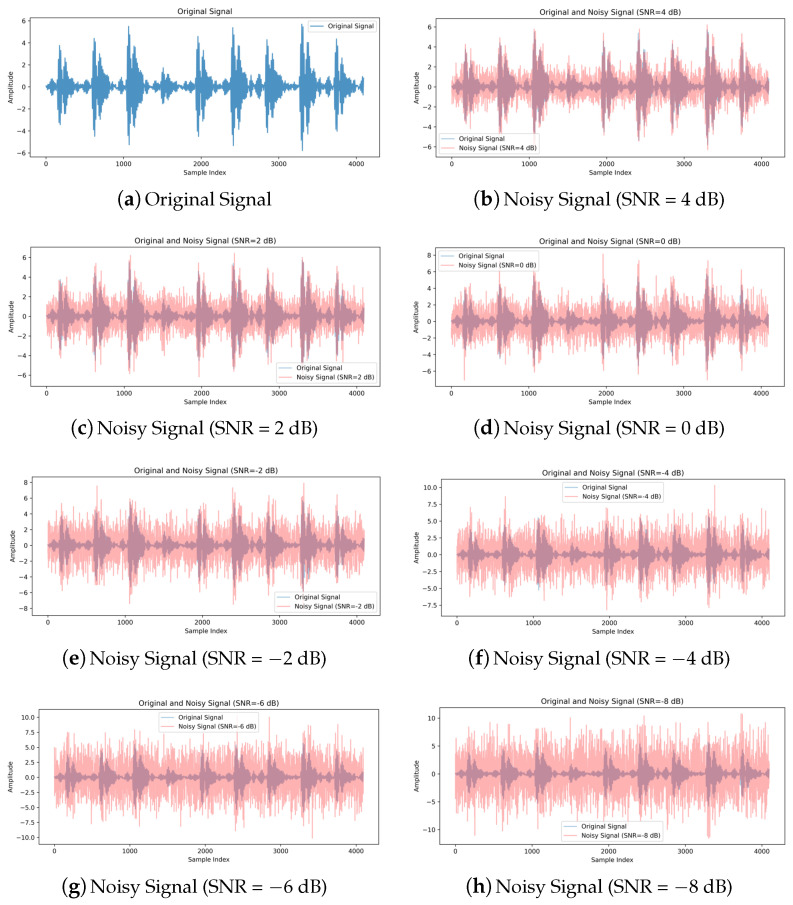
The comparison of the original signal and the noise signals.

SKF rolling bearing of model 6205-2RS is employed in Case Western Reserve University (CWRU) bearing dataset, which is provided by Case Western Reserve University in the United States. Vibration signals of the rolling bearing are collected at a sampling frequency of 48 kHz. The test platform is shown in Figure 7. Single-point faults are induced in the ball, inner raceway, and outer raceway, with fault diameters of 0.178 mm, 0.356 mm, and 0.532 mm, respectively. Therefore, the dataset contains 10 types of labels (9 for faults and 1 for normal). In order to facilitate feature extraction, the collected signals are subjected to min-max normalization to limit the preprocessed data within a certain range. The dataset is noise-free, and the current methods for solving fault diagnosis under noise environment are generally implemented by adding noise to the pure dataset. Therefore, the Gaussian white noise is used to simulate the interference in real scenes to verify the ability of the proposed method. The comparison of the original signal and the noise signals are shown in Figure 8. The equation for the SNR is shown in (23):

(23) $$SNR=10\log\left(\frac{P_{signal}}{P_{noise}}\right)$$

where $P_{signal}$ and $P_{noise}$ represent the effective power of the signal and noise, respectively.

#### 3.2.1. Comparison with Traditional Algorithms

**Figure 9:**
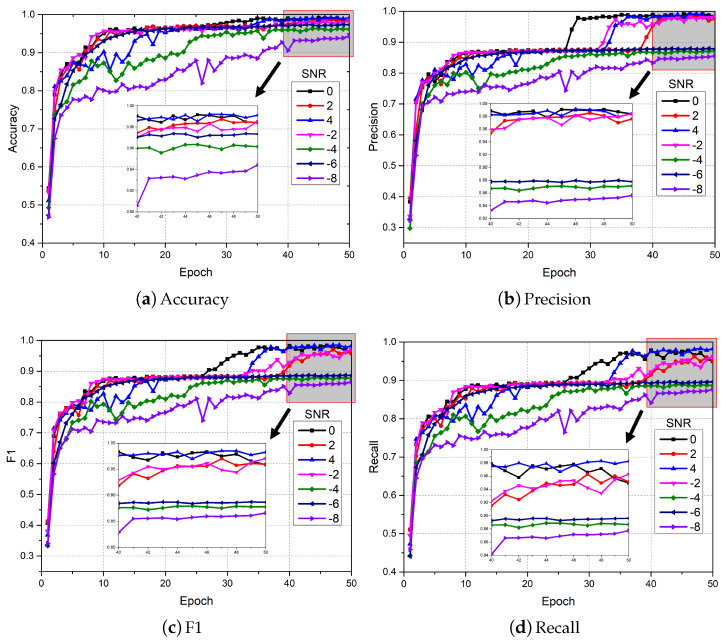
Diagnostic results of the proposed DTDNet under different SNRs.

In order to verify the effectiveness of the proposed DTDNet, five algorithms were used for comparative experiments, including 1D-CNN [37], 1D CNN with LSTM [38], 2D CNN with ELM [39], Pretrained Alexnet [40], deep convolutional neural networks with wide first-layer kernels (WDCNN) [41], and the state-of-the-art method JL-CNN [31]. The original vibration signal data were divided into 4096 segments and shuffled (a total of 9289); 80% of the data were used as training data, and the remaining data were used as test data. Optimization is performed using Adam with default parameters. The initial learning rate is set to $5\times 10^{-5}$, the batch size is set to 8, and the dropout rate is set to 0.5. The variation range of SNR in the experiment was set from −8 to 4 dB, with a step size of 2. The number of iterations of the experiment was set to 50. As the iteration proceeds, the changes in the four evaluation indicators for fault diagnosis using DTDNet are shown in Figure 9. The diagnostic accuracy of DTDNet is obviously affected by the noise in the data, but it still has excellent diagnostic capabilities for signals under different SNRs and ensures convergence performance.

The comparison results with other algorithms are shown in Table 2. 1D CNN is limited by feature extraction capability, and its diagnostic performance is much lower than that of other algorithms. By combining the long-range dependency information extraction ability of LSTM, 1D CNN with LSTM has better diagnostic accuracy than 1D CNN. Algorithms based on 2D CNN have the ability to capture more complex features. However, 2D CNN with ELM and Pretrained AlexNet are difficult to handle fault diagnosis tasks under low SNR, especially when the SNR is −8. WDCNN and JL-CNN have more advanced diagnostic performance, among which JL-CNN integrates fault diagnosis tasks and signal denoising tasks into an end-to-end CNN architecture, achieving good noise robustness through dual task joint learning. The proposed DTDNet can maintain excellent diagnostic performance under low SNR. It has 94.56% and 97.47% classification accuracy at −8 dB and −6 dB, respectively, which are 31.35% and 15.51% higher than the suboptimal algorithm JL-CNN. In all SNR experiments, DTDNet achieved the best diagnostic accuracy, which verifies the effectiveness of the method proposed in this paper. In addition, to compare the computational costs of different methods, we mainly compared the inference time of WDCNN, JLCNN, and the proposed method. The results indicate that the method proposed in this paper has good inference speed. For 4096 sequence samples, this method can provide diagnostic results at an average speed of 13.07 ms, which means that the sensor of the tested device can sample at frequencies greater than 300 kHz. Although WDCNN and JLCNN have good inference time results, the method proposed in this manuscript has significant advantages in diagnostic accuracy and can meet the needs of practical tasks.

#### 3.2.2. Ablation Analysis

**Figure 10:**
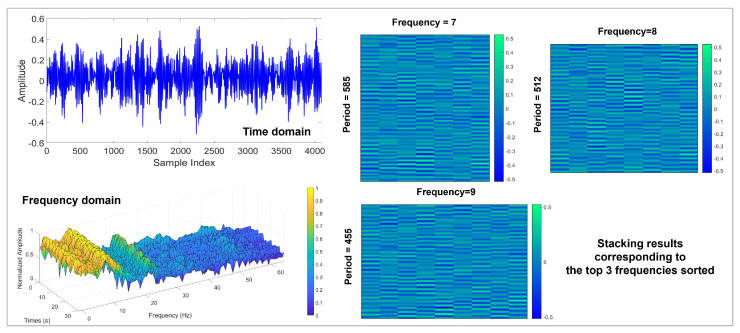
STFT decomposition on CWRU signals.

The schematic diagram of using STFT to decompose the CWRU signal is shown in Figure 10. The original signal is decomposed by STFT to obtain the top 3 sorted frequencies, and the signal segment is stacked based on the obtained 3 frequencies. To demonstrate the effectiveness of STFT, this work sets up 7 sets of experiments with SNR ranging from −8 to 4 dB and a step size of 2. The hyperparameter *k* (number of stacked images) ranges from 2 to 8 with a step size of 2. The experimental results are shown in Table 3. Applying STFT decomposition can effectively improve the diagnostic accuracy, especially when the *k* is large, which reveals the powerful ability of STFT to handle non-stationary signals. As the *k* increases, the model can capture more detailed information, which is beneficial to providing more accurate diagnostic results. When the *k* reaches 8, the average accuracy of the model in 7 sets of experiments is 97.69%, which is an improvement of 5.19% compared to applying FFT.

**Figure 11:**
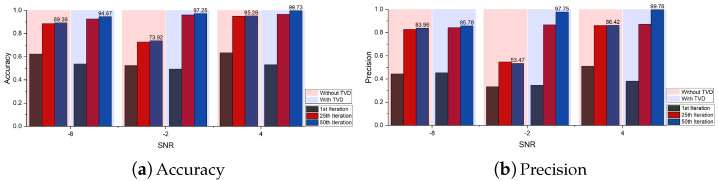

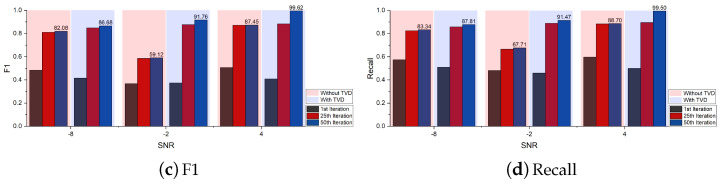
The ablation analysis results of TVD module.

**Figure 12:**
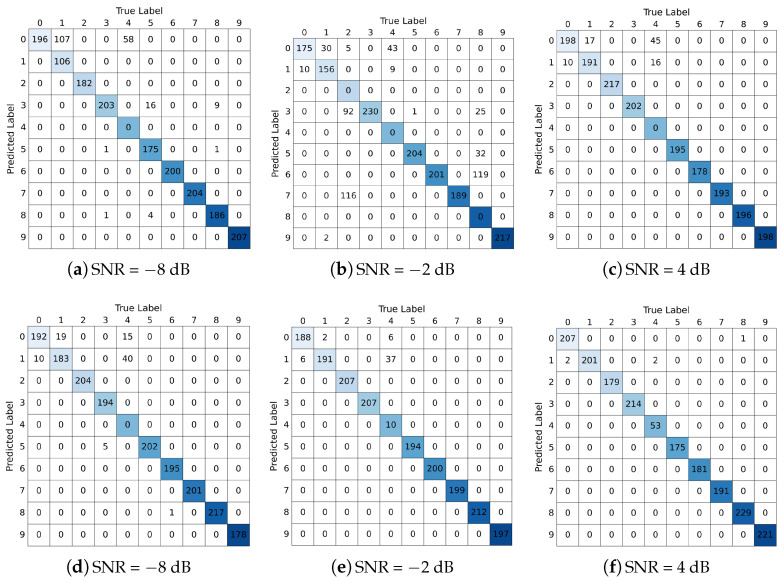
Ablation experiments on TVD modules under different SNRs. (TVD is not included in experiments (**a**–**c**). TVD is included in experiments (**d**–**f**)).

**Figure 13:**
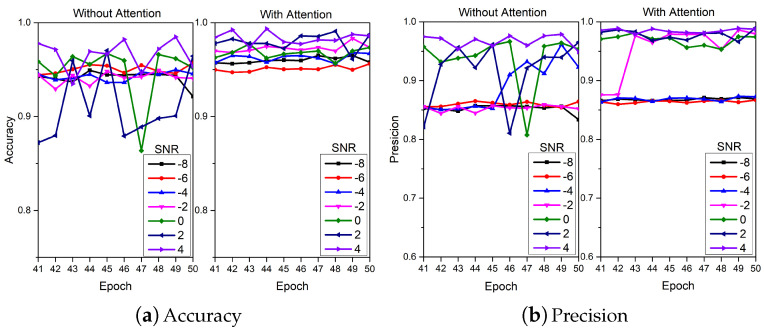

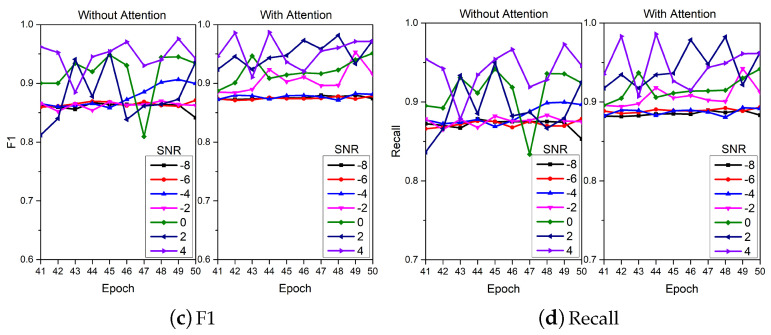
The ablation analysis results of MAF module.

The ablation analysis results of TVD and MAF modules are shown in Figure 11, Figure 12 and Figure 13 respectively. For the TVD module, this work sets up 3 sets of experiments at SNRs of −8, −2 and 4 dB. It can be clearly observed that adding the TVD module can effectively improve the diagnostic accuracy of the model, especially when the SNR is −2 dB (23.33% improvement in accuracy). In Figure 12, it can be observed that the diagnostic performance of the model is further improved after adding the TVD module, even when facing the problem of class imbalance (category label 4). For the MAF module, this work sets up 7 sets of experiments with SNR ranging from −8 to 4 dB and a step size of 2. Figure 13 shows the results from the 41st iteration to the 50th iteration. It can be found that by adding the MAF module, the model can achieve higher diagnostic accuracy and make the final accuracy change curve smoother.

#### 3.2.3. Comparison of Training Efficiency

**Figure 14:**
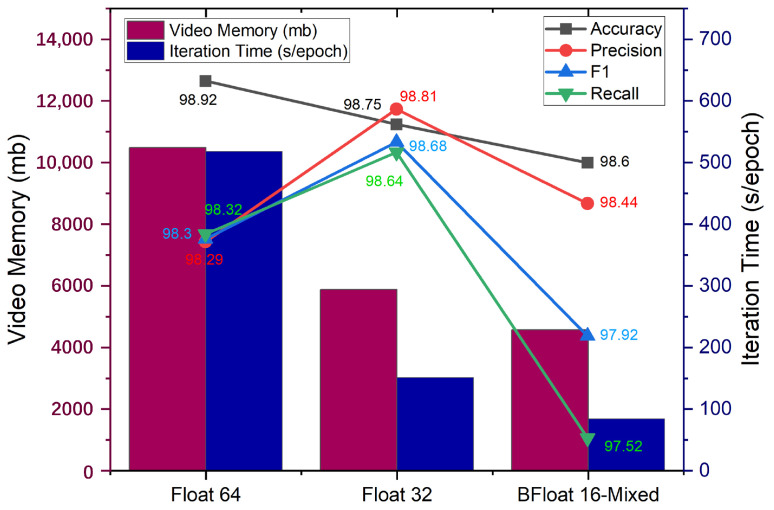
Results of different calculation strategies in terms of diagnostic accuracy, video memory consumption, and iteration time.

The optimization strategy of mixed precision calculation is applied to the training of DTDNet. The results of the model trained with 2 precisions of Float 32 and Float 64 in terms of diagnostic accuracy, video memory, and iteration time are shown in Figure 14. Applying mixed-precision calculations will slightly reduce the diagnostic accuracy of the model (0.12% of the Float 64), greatly reduce memory consumption (43.65% of Float 64) and iteration time (16.22% of Float 64), and improve the efficiency of training models. This is not a task-dependent strategy and can be embedded in the training of other deep learning models.

### 3.3. Real Aircraft Sensor Fault Dataset

#### 3.3.1. Dataset Description

**Figure 15:**
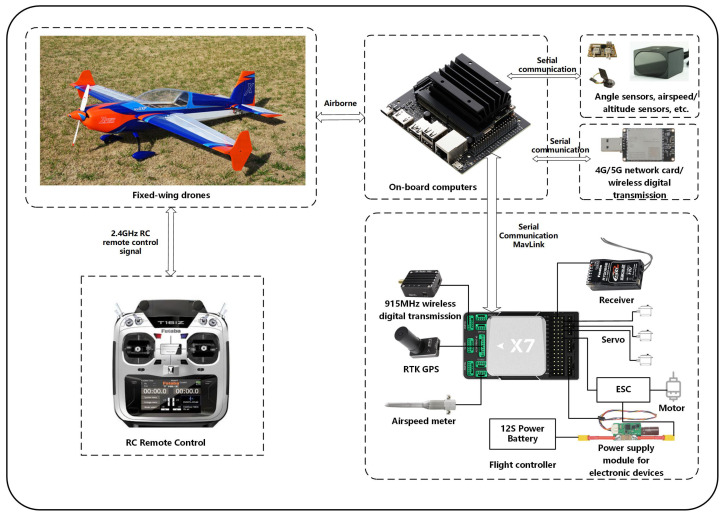
Configurations of the UAV adopted in this paper.

**Figure 16:**
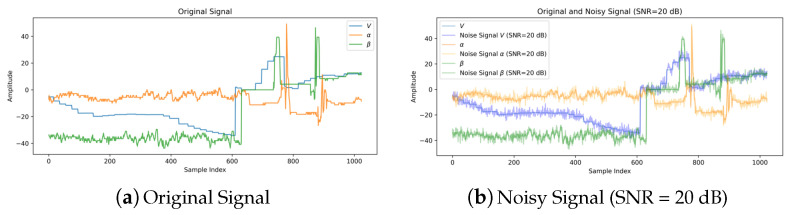

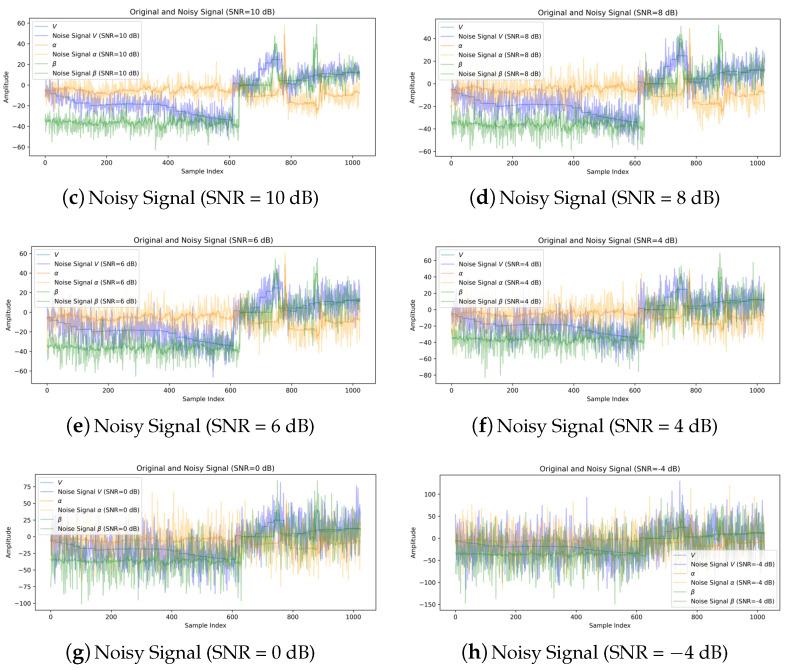
The comparison of the original signal and the noise signals.

In this study, this work conducted real aircraft data collection using a 78-inch EXTRA 300 NG (owned by Fudan University, Shanghai, China) fixed-wing unmanned aerial vehicle (UAV) equipped with the CUAV X7+ PRO (owned by Fudan University, Shanghai, China) flight control system, as depicted in Figure 15. This UAV is equipped with a range of sensors, including the CUAV C-RTK 9P GPS, SMV-1 non-contact Hall principle angle measuring vane (owned by Fudan University, Shanghai, China), and ADM800 altitude/airspeed meter (owned by Fudan University, Shanghai, China). Notably, many aviation accidents have been attributed to the Pitot tube becoming obstructed, leading to airspeed-related issues. Consequently, drift faults (manifesting as measurement loss) are taken into consideration for airspeed sensors ($V_{m}$). As for angle of attack (AOA, $\alpha_{m}$) and sideslip angle sensors ($\beta_{m}$), potential issues may arise from deflection vanes getting stuck or perturbed by external atmospheric conditions, giving rise to drift (constant bias) and additional noise faults. As detailed in Table 4, a total of five distinct fault cases are explored. By combining the normal data, this work has established a real aircraft sensor fault dataset that encompasses 6 categories. Gaussian white noise with different SNRs (−4, 0, 4, 6, 8, 10, and 20 dB) was added to the signals, and the results are shown in Figure 16.

#### 3.3.2. Comparison and Ablation Analysis

**Figure 17:**
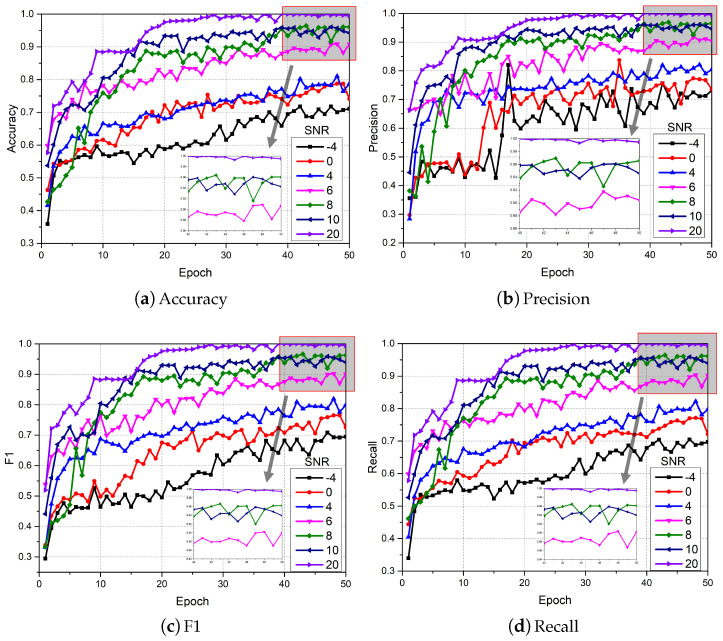
Diagnostic results of the proposed DTDNet under different SNRs.

**Figure 18:**
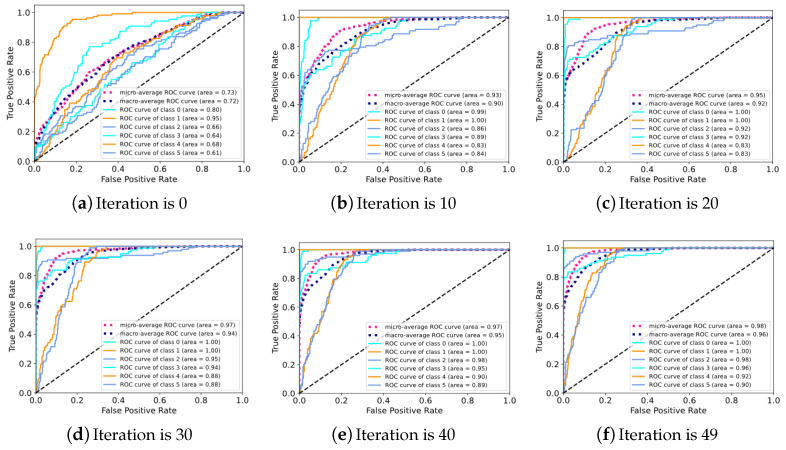
Fault diagnosis results of ROC curve.

**Figure 19:**
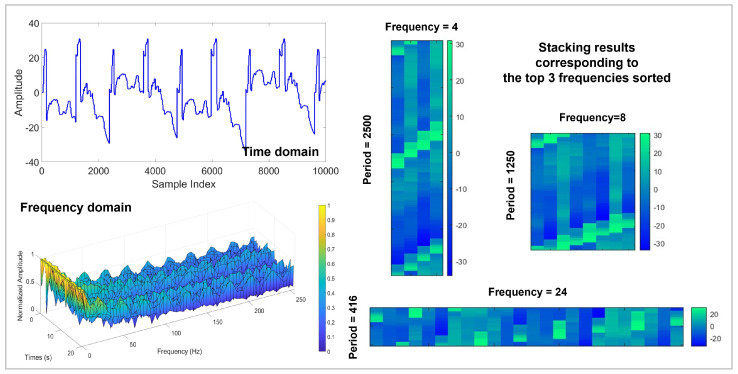
The STFT decomposition results on the real aircraft sensor fault signal.

On the Real aircraft sensor fault dataset, this work sets up experiments with 7 SNR conditions. The original vibration signal data was divided into 4096 segments and shuffled (a total of 11,028); 90% of the data was used as training data, and the remaining data was used as test data. Optimization is performed using Adam with default parameters. The initial learning rate is set to $5\times 10^{-5}$, the batch size is set to 8, and the dropout rate is set to 0.5. The number of iterations of the experiment was set to 50. As the iteration proceeds, the changes in the 4 evaluation indicators for fault diagnosis are shown in Figure 17. It can be clearly observed that compared to the results of the CWRU dataset in Figure 9, the performance of DTDNet shows a downward trend due to factors such as redundant information from multiple sensors and strong noise background. Especially when the SNR is 6, this work visualizes the ROC curve results of the model performing fault diagnosis as the iteration progresses, as shown in Figure 18, which demonstrate the excellent diagnostic results of the model for various categories of data. The schematic diagram of using STFT to decompose the aircraft sensor signal is shown in Figure 19, and it can be clearly observed in the stacking results that the information between cycles is captured. The results of the ablation experiments on STFT are shown in Table 5. Applying STFT can effectively improve the diagnostic accuracy of the model, especially when the *k* is 2 (a 3.52% improvement compared to FFT). Different from the results of the CWRU dataset, when the *k* value is too large (8), more interference information will be introduced, resulting in a decrease in the performance of the model. The comparison results with advanced methods WDCNN and JL-CNN and ablation analysis results for TVD and MAF modules are shown in Table 6. The method proposed in this paper achieves a 29.76% improvement compared to JL-CNN, verifying the effectiveness of the TDTNet. It can also be found that only adding the MAF module can bring a slight improvement in diagnostic accuracy to the model, especially at low SNR (−4). The role of the TVD module is even more significant, which verifies its effectiveness in performing signal processing in noisy environments.

## 4. Conclusions

To address noise-induced challenges in fault diagnosis of mechanical systems, this paper proposes the DTDNet model. Unlike the traditional approach of using denoising tools for noise reduction before using neural networks for fault diagnosis, which carries the risk of affecting important features in the signal and introduces some pseudofeatures, DTDNet uses a multi-scale convolutional network to extract features from the signal and then uses the TVD denoising module to perform non-linear processing on the features. Using STFT to decompose signals at multiple time resolutions is beneficial for the network to extract more important feature information and avoids the high constraints of FFT on signal stability. The inclusion of TVD and MAF modules further boosts diagnostic precision on both datasets, especially in low SNR conditions. Compared to existing methods such as 1D CNN, 1D CNN with LSTM, 2D CNN with ELM, Pretrained Alexnet, WDCNN, and JL-CNN, the proposed DTDNet demonstrates superior diagnostic accuracy. DTDNet has powerful noise reduction and feature extraction capabilities, and future work will focus on verifying the effectiveness of the model in other tasks, such as signal detection for current signatures and acoustic sound, and exploring its practical deployment through mobile devices, aiming to improve accessibility and effectiveness in different deployment environments.

## Figures and Tables

**Table 1:** Confusion matrix of predictions and labels.

	Predictions (Positive)	Predictions (Negative)
Labels (Positive)	TP	FN
Labels (Negative)	FP	TN

**Table 2:** The comparison experiments on CWRU.

Algorithm		SNR							Average
		−8	−6	−4	−2	0	2	4	
1D CNN [37]	Acc	14.72	18.45	26.31	33.97	39.11	43.25	48.49	32.04
	Pre	14.24	15.37	23.83	30.86	41.58	47.18	55.96	32.72
	Recall	14.45	18.25	26.47	34.49	39.46	43.93	49.24	32.33
	F1	14.25	15.95	24.59	31.67	38.39	42.05	48.24	30.74
1D CNN with LSTM [38]	Acc	17.54	30.85	40.63	49.09	56.45	70.16	81.25	49.42
	Pre	18.27	31.05	41.37	49.83	57.26	70.27	81.26	49.9
	Recall	17.80	31.70	41.41	50.46	57.92	71.55	82.25	50.44
	F1	17.93	31.20	41.13	50.05	57.49	70.68	81.50	50
2D CNN with ELM [39]	Acc	18.04	29.64	42.24	61.90	79.54	88.00	93.95	59.04
	Pre	19.29	30.29	43.01	62.89	80.63	88.62	94.42	59.88
	Recall	18.34	30.40	42.88	63.18	80.80	88.56	94.42	59.8
	F1	18.66	30.17	42.58	62.49	80.12	88.52	94.36	59.56
Pretrained AlexNet [40]	Acc	31.25	48.29	55.95	69.15	91.03	72.28	95.97	66.27
	Pre	38.43	54.57	64.84	72.61	92.73	84.59	96.31	72.01
	Recall	32.17	48.88	56.68	69.84	91.51	72.57	96.19	66.83
	F1	26.86	47.58	54.45	67.71	91.01	70.86	96.07	64.94
WDCNN [41]	Acc	30.65	68.15	77.42	77.52	97.38	87.50	98.59	76.74
	Pre	23.98	56.92	63.73	64.32	97.43	80.98	98.68	69.44
	Recall	31.26	69.60	79.17	79.16	97.56	88.79	98.63	77.74
	F1	23.51	61.84	70.14	70.40	97.44	84.28	98.65	72.32
	Time (ms)	0.111	0.109	0.112	0.103	0.108	0.105	0.103	0.107
JL-CNN [31]	Acc	63.21	81.96	93.15	90.52	96.88	99.40	99.90	89.29
	Pre	66.21	83.66	93.82	91.35	97.17	99.42	99.91	90.22
	Recall	64.69	82.71	93.64	91.39	97.14	99.41	99.90	89.84
	F1	65.23	83.08	93.69	91.34	97.15	99.41	99.91	89.97
	Time (ms)	0.21	0.212	0.206	0.204	0.209	0.209	0.203	0.208
Proposed DTDNet	Acc	94.56	97.47	96.66	97.79	99.25	98.76	99.35	97.69
	Pre	85.75	87.88	87.44	98.31	99.13	97.3	98.62	93.49
	Recall	87.86	89.71	89.14	94.37	97.76	96.27	98.63	93.39
	F1	86.71	88.75	88.2	95.55	98.36	96.71	98.63	93.27
	Time (ms)	12.98	13.26	13.2	12.97	12.96	13.16	12.99	13.07

**Table 3:** The diagnostic accuracy (%) of different decomposition methods on CWRU.

Algorithm	*k*	SNR							Average
		−8	−6	−4	−2	0	2	4	
FFT	2	89.71	90.19	91.11	90.41	93.05	94.34	91.81	91.52
	4	92.81	89.33	88.2	93.21	93.16	92.4	96.28	92.2
	6	90.52	91.65	92.35	94.23	96.23	94.72	92.73	93.2
	8	80.87	93.64	93.86	94.07	95.26	95.1	94.78	92.51
STFT	2	88.42	89.44	88.58	91.49	92.13	93.97	91.06	90.73 (↓ 0.52)
	4	94.45	94.07	95.37	94.83	94.23	96.55	94.94	94.92 (↑ 2.72)
	6	94.45	96.12	96.28	97.14	96.5	97.74	99.03	96.75 (↑ 3.55)
	8	94.56	97.47	96.66	97.79	99.25	98.76	99.35	97.69 (↑ 5.19)

**Table 4:** Aircraft sensors fault cases adopted in this paper.

Case	Sensor	Fault Type	Magnitude *
5	$\beta_{m}$	extra noise	$5^{\circ }$∼$10^{\circ }$
4	$\beta_{m}$	drift	$\pm (5^{\circ }$∼$10^{\circ })$
3	$\alpha_{m}$	extra noise	$5^{\circ }$∼$10^{\circ }$
2	$\alpha_{m}$	drift	$\pm (5^{\circ }$∼$10^{\circ })$
1	$V_{m}$	drift	−(50%∼100%)
0	clean measurement with noises and disturbances, no fault		

* Noise standard deviation and drift values defined in this column.

**Table 5:** The diagnostic accuracy (%) of different decomposition methods on real aircraft sensor fault dataset.

Algorithm	*k*	SNR							Average
		−4	0	4	6	8	10	20	
FFT	2	62.86	77.14	83.57	77.86	94.11	95.71	98.93	84.31
	4	67.32	59.11	78.93	81.43	94.11	88.57	99.82	81.33
	6	52.5	66.07	66.96	84.64	90.54	95.71	96.07	78.93
	8	54.11	68.04	75.54	88.75	90	93.57	94.11	80.59
STFT	2	71.79	78.75	81.07	90.89	96.43	96.07	99.82	87.83 (↑ 3.52)
	4	57.68	63.57	83.39	95.54	91.07	91.61	99.82	83.24 (↑ 1.91)
	6	55.54	63.75	80.89	87.86	88.57	91.43	99.82	81.12 (↑ 2.19)
	8	56.07	60.89	68.21	80.18	84.29	85.18	96.07	75.84 (↓ 4.75)

**Table 6:** The comparative and ablation experiments on real aircraft sensor fault dataset.

Algorithm		SNR							Average
		−4	0	4	6	8	10	20	
WDCNN [41]	Acc	33.69	37.73	40.95	45.45	44.26	45.17	68.01	45.04
	Pre	26.53	30.67	32.83	49.03	33.28	38.55	63.26	39.17
	Recall	33.67	38.01	40.81	45.73	43.94	45.29	68.24	45.10
	F1	28.67	31.26	34.01	40.69	35.87	37.60	63.23	38.76
JL-CNN [31]	Acc	43.47	46.78	54.14	57.81	61.12	64.15	70.50	56.85
	Pre	37.88	42.16	48.27	52.17	56.29	58.08	61.76	50.94
	Recall	43.47	46.82	54.16	57.64	60.83	64.67	70.54	56.88
	F1	38.70	42.82	49.34	53.20	57.09	59.62	65.02	52.26
Without TVD without MAF	Acc	52.14	58.04	71.25	71.79	89.11	88.57	98.75	75.66
	Pre	43.24	49.43	75.57	74.27	91.03	90.68	98.83	74.72
	Recall	54.15	58.27	73.35	71.21	89.75	88.26	98.66	76.24
	F1	46.96	52.81	71.9	71.85	89.51	88.9	98.71	74.38
Without TVD With MAF	Acc	56.07	71.07	78.04	84.47	83.93	92.14	99.82	80.79
	Pre	47.87	72.92	86.24	88.15	84.89	93.26	99.82	81.88
	Recall	58.87	72.64	79.19	85.31	84.16	92.87	99.81	81.84
	F1	49.05	71.8	76.62	84.67	84.42	92.07	99.82	79.78
With TVD Without MAF	Acc	68.57	75.71	82.86	85.89	91.78	94.82	98.57	85.46
	Pre	70.14	72.79	85.1	87.27	92.08	95.18	98.64	85.89
	Recall	69.18	73.31	83.45	86.18	91.69	95.17	98.5	85.35
	F1	69.49	72.52	83.06	85.54	91.49	95	98.48	85.08
With TVD With MAF	Acc	71.25	74.46	83.21	87.32	93.39	96.61	100	86.61
	Pre	66.46	72.78	84.69	88.96	94.64	96.69	100	86.32
	Recall	71.22	72.88	83.76	86.87	93.33	96.67	100	86.39
	F1	66.76	71.36	84.05	87.22	93.6	96.62	100	85.66

## Data Availability

Data are available on request from the authors.
